# Short‐Term Impacts of PM_2.5_ Components on Schizophrenia Hospitalization: A Time‐Series Analysis From Nanning, China

**DOI:** 10.1029/2025GH001556

**Published:** 2026-06-07

**Authors:** Zukang Gong, Baihua Chen, Bin Xu, Lan Lan, Shuiqing Qin, Li Su, Wenzhen Lin, Jianxiong Long

**Affiliations:** ^1^ The Fifth People's Hospital of Nanning City Nanning China; ^2^ School of Public Health Guangxi Medical University Nanning China; ^3^ Nanning Center for Disease Control and Prevention Nanning China; ^4^ China (Guangxi) ‐ ASEAN Engineering Research Center of Big Data for Public Health Guangxi Medical University Nanning China; ^5^ Department of Biochemistry and Molecular Biology School of Basic Medical Sciences Guangxi Medical University Nanning China

## Abstract

Schizophrenia (SCZ) is a serious psychiatric condition. While PM_2.5_ exposure has been linked to SCZ, the specific effects of its components remain poorly understood. This study aimed to explore the relationships between PM_2.5_ constituents (including BC: black carbon, OM: organic matter, SO_4_^2−^: sulfate, NH_4_^+^: ammonium, and NO_3_^−^: nitrate) and SCZ. It incorporated the hospitalization records of 16,082 SCZ patients from Nanning Fifth People's Hospital, spanning from 1 January 2014, to 31 December 2023. The daily concentration data of PM_2.5_ and its five chemical components were sourced from Tracking Air Pollution in China (TAP). A distributed lag nonlinear model (DLNM) was employed to measure the dynamic correlation between PM_2.5_ components and the risk of hospitalization for SCZ. Further analysis was conducted by stratifying based on gender, age, and cold/warm seasons to identify susceptible populations. Our study revealed that OM and BC demonstrated lagged effects on SCZ hospitalization, with significant associations observed at lag 3 day(lag3)and lag4. The strongest effect was identified at lag4, with OM showing an relative risks (RR) of 1.010 (95%CI: 1.001, 1.019) and BC exhibiting a higher RR of 1.010 (1.001, 1.019). And the lag effect of the OM relative percentage was identified at lag 3 (RR = 1.013, 95% CI: 1.005–1.022). PM_2.5_, SO_4_^2−^, and NH_4_^+^ showed lagged response trends but no statistically significant effects (*p* > 0.05). Subgroup analysis indicated that males, 45 years and younger, and those exposed during the warm season had higher risks associated with SO_4_^2−^, OM, BC, and PM_2.5_. Short‐term exposure to OM is significantly related to SCZ hospitalization.

## Plain Language Summary

PM_2.5_ is a recognized risk factor for schizophrenia, yet its underlying mechanisms remain unclear. The toxicological properties of PM_2.5_ are governed by its components, but systematic studies on their roles in schizophrenia are scarce. This study investigates the impact of PM_2.5_ and its five major chemical components on schizophrenia hospitalization, aiming to identify specific components contributing to the disease and provide evidence for source‐specific PM_2.5_ pollution control.

## Key Points

- PM_2.5_ components of OM and BC exhibit lagged effects on the risk of schizophrenia hospitalization
- Males, middle‐aged and young patients with schizophrenia, and those exposed during the warm season face a higher risk of hospitalization

## 1 Introduction

Schizophrenia (SCZ) is a serious mental disorder. Its core symptoms are hallucinations, delusions, and affective flattening (McCutcheon et al., 2020). Globally, around 24 million people suffer from SCZ, with a lifetime prevalence ranging from 0.3% to 0.7%. According to the GBD (GBD, 2022), the crude prevalence, incidence, and disability–adjusted life years have risen by 65%, 37%, and 65% respectively. While genetic and early‐life factors are regarded as the main causes of SCZ (Jauhar et al., 2022), in recent years, the influence of environmental exposures on the development and progression of SCZ has increasingly drawn attention (Remme et al., 2021).

Experimental investigations found that PM_2.5_ particulates can traverse the blood‐brain barrier via two primary pathways—the olfactory neural route (Hopkins et al., 2018) or systemic circulatory system (Ha et al., 2022)—thereby inducing potential damage in the central nervous system. Research across various regions has demonstrated a positive correlation between PM_2.5_ exposure and the risk of hospitalization for schizophrenia (Bai et al., 2024; Nguyen et al., 2021). However, studies on short term particulate matter exposure and SCZ admissions often exhibit considerable heterogeneity in lagged responses and subgroup analysis outcomes (Li et al., 2020; Song et al., 2018). This variability may be attributed to differences in PM_2.5_ chemical composition (Masselot et al., 2022).

The chemical components of PM_2.5_ primarily include black carbon (BC), organic matter (OM), sulfate (SO_4_^2−^), ammonium (NH_4_^+^),nitrate (NO_3_^−^) and other trace elements and metals. Identifying these chemical components is of great significance for tracing the sources of PM_2.5_ and exploring the specific toxicological mechanisms (López‐Martín et al., 2024; Wang et al., 2022). BC can induce oxidative stress, neuroinflammation (Shang et al., 2019), and mitochondrial dysfunction (Shang et al., 2022). The toxicological effects of organic matter (OM) in PM2.5 are composition‐dependent, with polycyclic aromatic hydrocarbons (PAHs) identified as a critical risk factor. PAHs exert toxicity primarily through two mechanisms: (a) DNA damage via metabolic transformation (Stading et al., 2021) and (b) OGG1 gene promoter methylation (Fu et al., 2023). Perfluorinated compound (PFAS) have emerged as a novel class of contaminants detected in the OM fraction of PM_2.5_ in recent years (Li et al., 2022), though their toxicological mechanisms remain poorly characterized. NH_4_^+^ is considered neurotoxic under conditions of high concentration exposure or metabolic abnormalities (Heidari, 2019). SO_4_^2−^ and NO_3_^−^ are regarded as low‐toxicity substances, requiring further experimental evidence to confirm their toxicity. Epidemiological studies have linked PM_2.5_ components to dementia (Shi et al., 2023), and depressive symptoms (Ji et al., 2024). To our knowledge, a sole investigation exists on the correlation between PM_2.5_ constituents and SCZ admissions (Pan, Song, et al., 2024), which is from the Anhui region of China.

To examine the differential effects of PM_2.5_ chemical species on schizophrenia‐related hospitalization risks and their temporal patterns, this study hypothesizes that PM_2.5_ chemical species exert component‐specific effects with distinct lagged patterns. By applying the Distributed Lag Nonlinear Model (DLNM), this study will dynamically quantify the impacts of PM_2.5_ chemical species, capturing the temporal variability in exposure‐response relationships and identifying component‐specific risks. Stratified analyses by gender, age, and season will be conducted to identify vulnerable populations, thereby informing precise source control strategies and population‐specific protective measures.

## 2 Methods

### 2.1 Data Collection

As the administrative hub of Guangxi Zhuang Autonomous Region in southwestern China, Nanning encompasses 22,100 km^2^ of territorial jurisdiction and maintained a 2023 resident population of 8.94 million. We sourced case data on schizophrenia spanning 1 January 2014, to 31 December 2023, from the Fifth People's Hospital of Nanning. This hospital is the city's only tertiary class a psychiatric specialist hospital and its largest psychiatric institution. The case records included details like admission dates, patients' ages, genders, patient numbers, and ICD‐10 (International Classification of Diseases, 10th Revision) discharge diagnosis codes. All patients with a primary discharge diagnosis of SCZ (ICD‐10: from F20.0 to F20.9) were included in the study. The data were reviewed, non‐local residents were systematically excluded based on verified residency parameters.

Daily data of PM_2.5_ and its five chemical components (including PM_2.5,_ SO_4_^2−^, NO_3_^−^, NH_4_^+^, OM, BC) from 1 January 2014 to 31 December 2023 in Nanning were derived from Tracking Air Pollution in China (http://tapdata.org.cn/?page_id=59&item=pm25). TAP, a data set of atmospheric aerosol composition with high spatial‐temporal accuracy, was constructed using machine learning algorithms and multi‐source data (Liu et al., 2022; Xiao et al., 2021). According to previous reports (Su et al., 2024), we calculated the average value of all grids within the Nanning range, representing the exposure level in the geographical area. This method can effectively cover the sparse areas of monitoring sites. Meteorological parameters, including daily mean temperature (MT, °C) and relative humidity (RH, %), were sourced from China Meteorological Data Service Center (https://data.cma.cn/). This study was approved by the ethics committee of Fifth People's Hospital of Nanning (SL2024‐41‐01).

### 2.2 Statistical Analysis

PM_2.5_ has a non‐linear and lagged effect on individuals with mental disorders (Liu et al., 2023). This study investigated associations between specific PM_2.5_ constituents and schizophrenia‐related hospital admissions, this study used a combination of the generalized linear model (GLM) with DLNM.

Figure S1 in Supporting Information S1 shows that the five chemical compositions of PM_2.5_ and meteorological factors exhibit specific temporal distribution patterns. To control for secular trends and periodic fluctuations inherent in hospitalizations, Natural cubic splines (ns) were employed. According to previous studies (Liu et al., 2023), the degrees of freedom (df) for the time trend were set at 7 per year, and 3 df were assigned to MT and RH respectively. Day of the week (DOW) was represented by a categorical variable coded as integers from 1 to 7, while statutory holidays were defined as a dichotomous variable (0 = non‐holiday, 1 = statutory holiday); both variables were included in the model to adjust for the effects of weekends and statutory holidays. The analytical model applied in this study is described below:

$$\mathrm{Log}\left(Y_{t}\right)=\alpha +\text{cb}\left(\text{Xt},\text{lag}\right)+\text{ns}\left(\text{Temperature},3\right)+\text{ns}\left(\text{Humidity},3\right)+\text{ns}\left(\text{Time},7/\text{year}\right)+\text{Factor}\left({\text{DOW}}_{t}\right)+\text{Factor}\left(\text{Holiday}\right)$$

In the equation, (Y_t_) represents the expected value of SCZ on day t. Here, α serves as the intercept, and X_t_ is the average concentration of PM_2.5_ chemical constituents on day t. The cross‐basis function (cb) was deployed to quantify the temporal lag effects of PM_2.5_ and its constituent‐specific exposures on schizophrenia‐related hospitalization outcomes. The lag‐response window for exposure‐outcome dynamics was defined as 7 days. This implies that the model took into account the impact from lag 0 to lag 7 after PM_2.5_ and its component concentrations exposure. TIME represents each day in the series and functions to eliminate the long‐term trends and seasonal effects. Additionally, DOW and Holiday were incorporated to consider holiday effects. Considering the high collinearity between PM_2.5_ constituent concentrations and total mass concentration, two supplementary analyses were employed to disentangle constituent‐specific effects on SCZ hospitalization risk: (a) inclusion of total PM_2.5_ as a covariate in single‐constituent regression models (Su et al., 2024), and (b) development of compositional models based on the relative proportion of each constituent with the total PM_2.5_ concentration fixed.

To assess the levels of PM_2.5_ and the concentrations of its components affects different populations, we categorized the subjects by gender, age (divided into <45 years and ≥45 years groups), and season (the warm season spanning from May to October and the cold season from November to April). The age stratification was based on the high‐prevalence age ranges for schizophrenia (Charlson et al., 2018; Ji et al., 2022). Furthermore, the degrees of freedom of TIME were adjusted within the range of 5–9, those of MT within 1–5, and those of RH within 1–5. This adjustment was intended to ensure the robustness of the model in analyzing the correlation between PM_2.5_ and its components exposure and hospital admission for SCZ.

The same model and parameters were used for each fitting process. The results are shown as relative risks (RR) along with 95% confidence intervals (95% CI). The results are shown as relative risks (RR) along with 95% confidence intervals (95% CI). These values represent the impact on hospitalization of SCZ for per IQR increase n the concentrations or relative proportions.

## 3 Results

### 3.1 Demographic and PM_2.5_ Components Exposure Characteristics

From 1 January 2014, to 31 December 2023, a total of 16,082 admissions of patients with SCZ met the inclusion and exclusion criteria. Among the SCZ hospitalizations, there were 8,228 male patients (51.16%), and 7,854 female patients (48.84%), and most of them were younger than 45 years old (11,601, 72.14%). The number of patients hospitalized for SCZ in the cold season (8,535, 53.07%) was slightly higher than that in the warm season (7,547, 46.93%). The concentrations (mean ± SD) of PM_2.5_, SO_4_^2−^, NH_4_^+^, NO_3_^−^, OM, BC in Nanning City were 28.14 ± 17.09, 5.63 ± 3.23, 4.07 ± 3.11, 5.17 ± 4.27, 7.87 ± 4.84, 1.54 ± 0.92 (μg/m^3^), respectively. The values (mean ± SD) of MT and RH in Nanning City were 27.58 ± 6.32 (°C) and 79.39 ± 12.16 (%), respectively. The detailed results are shown in Table 1.

**Table 1:** Summary Statistics of Daily SCZ Admissions, Levels of PM_2.5_ Components, and Meteorological Factors in the Fifth People's Hospital of Nanning, 2014–2023

Variables	N	Mean ± SD	Min	P25	P50	P75	Max
SCZ	16,082	–	0	3	4	6	19
Gender							
Male	8,228	–	0	1	2	3	14
Female	7,854	–	0	1	2	3	11
Age							
<45	11,601	–	0	2	3	4	15
≥45	4,481	–	0	0	1	2	11
Warm and cold season							
Warm	7,547	–	0	3	4	5	14
Cold	8,535	–	0	3	4	6	19
PM2.5 and its components							
PM_2.5_ (μg/m^3^)	–	28.14 ± 17.09	1.00	15.93	24.3	36.68	114.09
SO_4_^2−^ (μg/m^3^)	–	5.63 ± 3.23	0.16	3.30	4.94	7.41	25.65
NH_4_^+^ (μg/m^3^)	–	4.07 ± 3.11	0.13	1.68	3.19	5.60	19.92
NO_3_^−^ (μg/m^3^)	–	5.17 ± 4.27	0.138	2.21	3.80	6.79	31.44
OM (μg/m^3^)	–	7.87 ± 4.84	0.20	4.50	6.88	10.05	34.57
BC (μg/m^3^)	–	1.54 ± 0.92	0.03	0.90	1.35	1.98	6.91
Weather factors							
MT (°C)	–	27.58 ± 6.32	3.80	17.50	23.60	27.58	32.30
RH (%)	–	79.39 ± 12.16	25.00	74.00	82.00	87.00	100.00

*Note.* SCZ, Schizophrenia; SO_4_^2−^, sulfate; NO_3_^−^, nitrate; NH_4_^+^, ammonium; OM,organic matter; BC, carbon black.

### 3.2 PM_2.5_ and Its Components on SCZ Admissions

**Figure 1:**
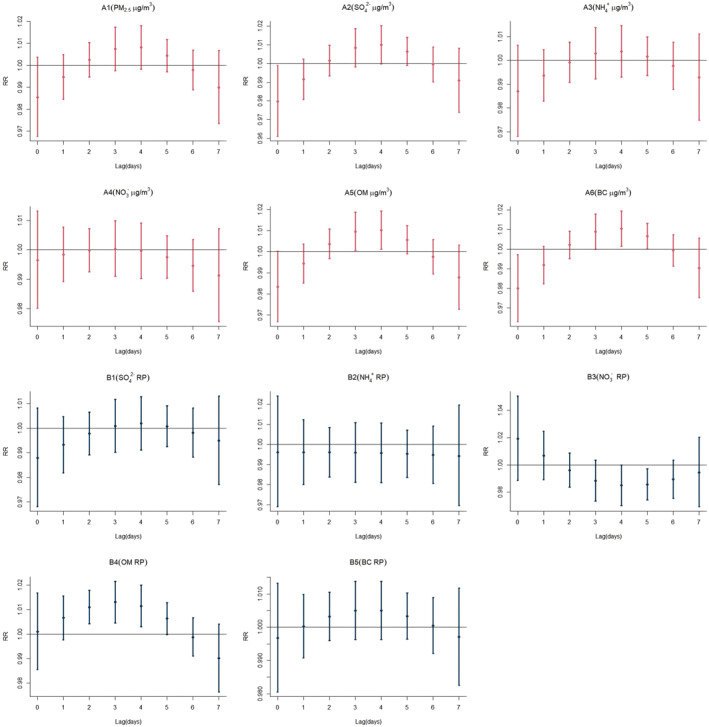
Relative risk (RR) and 95% CI for the relationship between PM_2.5_ components exposure and their relative proportions and SCZ hospitalization. Note: 1. RP: relative proportions, representing changes in the relative proportions of PM2.5 components. 2. RR represents changes in SCZ hospitalization risk per inter‐quartile range (IQR) increase in PM_2.5_ components (A1–A6) or its RP (B1–B5).

Figure 1, Table S2, and Table S3 in Supporting Information S1 summarize the impact of atmospheric PM_2.5_ and its five components on hospitalization for SCZ. In the context of this study, lag0‐lag7 represents different time‐lag periods. The RR (95%CI) of OM was observed from lag 3 days and lag 4 days, with 1.010 (1.001, 1.019). The effect of BC appeared at lag4, and the RR (95%CI) was 1.010 (1.001, 1.019). Although PM_2.5_, SO_4_^2−^ and NH_4_^+^ showed a lag response trend, with the highest effects at lag3 or lag4, no significant effects were actually detected for these, and a protective effect of SO_4_^2−^ and BC at lag 0 was noted, which disappeared with prolonged lag time. For the relative change model, the single‐day lag effects of OM RP from lag 2 to lag 4 showed statistically significant positive associations with SCZ hospitalization, with the strongest effect observed at lag 3 (RR = 1.013, 95% CI: 1.005–1.022). Conversely, an increase in the RP of NO_3_^−^ was associated with a reduced risk of SCZ hospitalization at lag 4 (RR = 0.987, 95% CI: 0.977–0.998).

### 3.3 Subgroup Analysis Results

**Figure 2:**
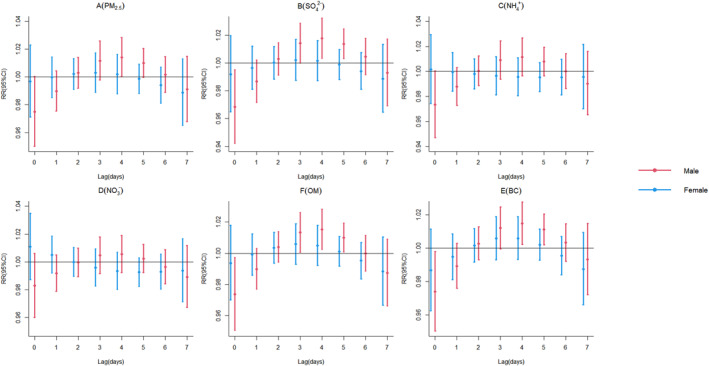
Single day lag effects of PM_2.5_ and its five components, stratified by gender. Note: The RR of SCZ hospitalization was calculated per IQR increase in PM_2.5_ concentration for lag 0–7 days.

Stratified analysis by gender groups is shown in Figure 2 and Table S4 of Supporting Information S1. In males, we observed significant effects on SCZ of SO_4_^2−^, BC, OM. However, no such significant effects were detected in females. In males, significant RR (95%CI) were identified between incident SCZ and SO_4_^2−^ at lag4 (RR = 1.018, 1.004–1.032) and lag5 (RR = 1.014, 1.003–1.025); BC at lag4 (RR = 1.015–1.002) and lag5 (RR = 1.011, 1.002 –, 1.020); and OM at lag3 (RR = 1.013, 1.001–1.026), lag4 (RR = 1.015, 1.003–1.028), lag5 (RR = 1.010, 1.001–1.019). Significant effects were found for neither NH_4_^+^ nor NO_3_^−^, whether in male or female.

**Figure 3:**
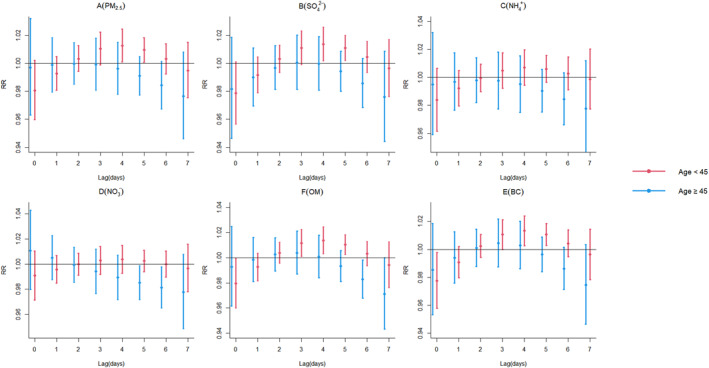
Single day lag effects of PM_2.5_ and its five components, stratified by age group. Note: The RR of SCZ hospitalization was calculated per IQR increase in PM_2.5_ concentration for lag 0–7 days.

As for the age group analysis (Figure 3 and Table S5 in Supporting Information S1), PM_2.5_, SO_4_^2−^, BC, and OM had an impact on SCZ among people of 45 years and younger, but not among 45 years and older. Their largest effects were observed at lag 4 day. The largest significant (95%CIs) of SO_4_^2−^, OM, BC and PM_2.5_ were 1.014 (1.002–1.026), 1.014 (1.003–1.025), 1.013 (1.003, 1.024) and 1.013 (1.001–1.025), respectively.

**Figure 4:**
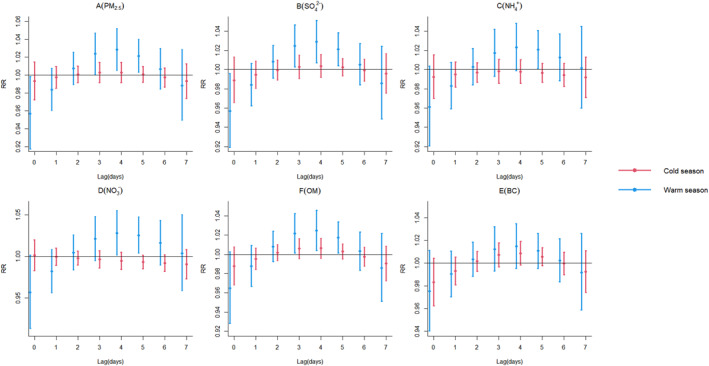
Single day lag effects of PM_2.5_ and its five components, stratified by season. Note: The RR of SCZ hospitalization was calculated per IQR increase in PM_2.5_ concentration for lag 0–7 days.

The stratified results by season are presented in Figure 4 and Table S6 of Supporting Information S1. Specifically, PM_2.5_ effects spanned from lag3 to lag5, peaking at 1.028 (1.005–1.052) on lag4. SO_4_^2−^ had lag effects on lag3 and lag4, peaking at 1.029 (1.007–1.051) at lag4. NH_4_^+^ had lag effects at 1.021 (1.001–1.040) on lag5. NO_3_^−^ effects were on lag4 and lag5, peaking at 1.028 (1.002–1.055 on lag4, and OM's single‐day lag effects, present on lag3, lag4, and lag5, also peaked at 1.025 (1.004–1.046) on lag4. However, BC did not show any significant effect in the seasonal stratification.

### 3.4 Sensitivity Analysis

In the sensitivity analyses, we found that when additionally adjusting for total PM_2.5_, the effect of NO_3_^−^ attenuated over time, although the effect was not statistically significant. The associations between the other four PM_2.5_ constituents and the risk of SCZ hospitalization showed minor changes but no substantial alterations (Figure S2 in Supporting Information S1). Similarly, models adjusting the degrees of freedom for average temperature, relative humidity and time yielded robust results consistent with those of the main model (Tables S7 and S8 in Supporting Information S1).

## 4 Discussion

This research delves into the potential link between brief exposure to the five chemical components of PM_2.5_ and hospitalizations for schizophrenia. DLNM were used to analyze the lagged effects of PM_2.5_ components concentration levels and their relative proportions on SCZ hospitalization risk. In the time‐series study conducted in Nanning, we found that organic matter concentrations and increased the risk of SCZ hospitalization. Subgroup analysis showed that males, people of 45 years and younger and subjects in the warm season were more sensitive to the exposure of SO_4_^2−^, OM, BC and PM_2.5_. In addition, no significant association between NH_4_^+^, NO_3_^−^ and SCZ hospitalization was found in this study.

Several studies have revealed that short‐term exposure to PM_2.5_ increases the risk of SCZ hospitalization, but research on PM_2.5_ components remains limited. A study in Anhui Province, China, indicated that five components elevated the likelihood of SCZ hospitalizations. BC raised the risk of SCZ admission by 1.59% (95% CI: 0.95%–2.23%), succeeded by sulfate (0.57%, 95% CI: 0.42%–0.72%), ammonium (0.52%, 95% CI: 0.37%–0.68%), organic matter (0.35%, 95% CI: 0.23%–0.46%). Significantly, these components had a more pronounced impact compared to PM_2.5_ itself (Pan, Song, et al., 2024). Considering the limited research systematically investigating the relationship between PM_2.5_ components and schizophrenia, this study also takes into account the reports on depression and degenerative diseases. A study covering the scope of China revealed that SO_4_^2−^ (OR = 1.287, 95% CI: 1.164–1.422) and OM (OR = 1.417, 95% CI: 1.245–1.612) were linked to depression outpatient visits during short–erm exposure. However, the correlation of these components was less strong than that of PM_2.5_ (Zhuang et al., 2024). A Chinese study demonstrated an association between PM_2.5_ (RR = −0.876, 95% CI: −1.205 to −0.548), organic carbon (RR = − 0.481, 95% CI: −0.744 to −0.219) and cognitive function (Pan et al., 2022). PM_2.5_ components are also associated with neurodegenerative diseases, and among them, the BC (OR = 1.81, 95% CI: 1.45–2.27) effect has been found to be the strongest (Wu et al., 2024). These studies are consistent with the current study in that exposure to PM_2.5_ components may increase the risk of psychiatric disorders or neurodegenerative diseases, and BC, OM, and SO_4_^2−^ often show stronger associations. However, current reports present some contradictory results. Unfortunately, this study did not observe significant connections between PM_2.5_ and other components (excluding BC and OM) and SCZ hospitalization in the overall study population, which may be due to exposure levels in the study area, demographic characteristics of the study subjects, and different study designs.

We found that an increase in the proportion of OM in PM2.5 also elevated the hospitalization risk of SCZ in the overall study population; in contrast, no such association was observed for BC. This seems to indicate that the effect of OM is independent of the total mass concentration of PM_2.5_, but is determined by its own content and compositional characteristics (Wang et al., 2025). In addition, the proportion of nitrate exhibited a certain protective effect at lag 5, which may be attributed to the source competition relationship between components (Masselot et al., 2022). Specifically, although both nitrate and OM are regarded as traffic‐related components, nitrate is mainly derived from the combustion of fuel oil and gas, while OM additionally originates from the combustion of various biofuels (McDuffie et al., 2020). This is an interesting finding, which provides evidence for exploring the focus of emission reduction strategies. That is, compared with controlling other PM2.5 sources, strengthening the control of biomass and biomass fuels may exert greater health benefits for SCZ patients.

In subgroup analysis, we found that males, young and middle‐aged adults (45 years and younger), and individuals in warm seasons demonstrated stronger associations with PM_2.5_ and its components. A study by Huo Liu et al. in northeastern China reported higher sensitivity to PM_2.5_ for SCZ among females (RR = 1.014, 95% CI: 1.002–1.025), aged 18–60 years (RR = 1.040, 95% CI: 1.001–1.082) and individuals in cold seasons (RR = 1.020, 95% CI: 1.009–1.032) (Liu et al., 2023). Coastal Chinese studies showed that young people were more susceptible to PM_2.5_ than the elderly, and males more than females (Ji et al., 2021). Gender differences in susceptibility to particulate matter exposure might arise from biological sex differences or/and gender roles, yet the underlying mechanisms remain poorly understood (Silveyra et al., 2021). Additionally, behavior and exposure patterns may be the reasons why young and middle‐aged adults as well as males are more sensitive (Peters et al., 2024). A comprehensive review indicates that most studies have reported peaks in SCZ hospitalization rates during summer, along with a positive association between high temperature (>28°C) and SCZ (Jahan et al., 2020), which aligns with the seasonal grouping results of this study. During the warm season, the effect of heatwaves may increase the risk of hospital admission for those with SCZ (ER% = 7.26%. 95% CI: 4.45%–10.14%) (Hu et al., 2023). Collectively, these divergent findings highlight the complexity of the relationship between PM_2.5_ components and mental disorders and underscore the need for further exploration.

This study found positive associations between short‐term exposure to BC, OM, and SO_4_^2−^ components of PM_2.5_ and SCZ hospitalizations, while no significant relationships were observed for NH_4_^+^ or NO_3_^−^. Mechanistically, short‐term exposure may mediate its effects via inflammation induction, respiratory and ocular irritation, tachycardia, and mood disturbances–pathophysiological states known to be mechanistically linked to SCZ (Al‐Kindi et al., 2020; Tong et al., 2024; Zhu et al., 2024). Neuroinflammation and oxidative stress are recognized as critical mechanisms underlying PM_2.5_‐mediated mental disorders (Hussain et al., 2023), though their precise pathways remain unclear. This process may involve microglia, the brain's sentinel cells (Kwon & Koh, 2020). Upon direct or indirect stimulation by particulate matter, microglia may become activated, triggering the MAC1‐NOX2 pathway associated with microglia‐mediated neurotoxicity (Jayaraj et al., 2017).

BC not only exerts direct pro‐inflammatory effects but also serves as a carrier for other components, establishing it as a critical constituent in mediating PM_2.5_ toxicity (López‐Martín et al., 2024); The acidic irritation of SO_4_^2−^ induces oxidative stress and inflammatory responses in the body (Wang et al., 2024); and water‐soluble components such as NO_3_^−^ and NH_4_^+^ undergo rapid metabolism and renal excretion, thereby minimizing systemic damage (Deng et al., 2016), this may account for the non—significant findings of nitrate and ammonium salts in this study. Additionally, there are complex interactions among the components. Although most OM is adsorbed onto BC (Chen et al., 2023), these two components compete for absorption sites in vivo, potentially leading to overestimation of OM's standalone effects (Ju et al., 2023). Meanwhile, BC‐SO_4_^2−^ synergy enhances sulfate penetration into alveolar macrophages, thereby potentiating oxidative stress and inflammatory responses (Peralta et al., 2021).

This study in Nanning city indicated the association between black carbon and organic matter and SCZ, identifying their distinct impacts on the hospitalization risks of SCZ patients, thereby providing evidence for targeted air pollution control strategies. Different from previous studies, we found that the impact of OM is robust and independent of total PM_2.5_ mass concentration. Combined with the characteristics of local emission sources in Nanning, this suggests that at the level of pollutant source control, it is necessary to strengthen the management of organic matter emission sources; in addition to conventional traffic emissions and industrial emissions, straw combustion is an important contributor to OM in this region (Pan et al., 2023). Additionally, the study identified susceptible populations for PM_2.5_ components, offering support for tailored protective measures. Personal protection should be strengthened, with special attention paid to males, young people and populations exposed in warm seasons; considering that SCZ patients spend a long time at home, it is a priority to recommend the installation of air purifiers to reduce the impact of indoor pollution (Pan, Yi, et al., 2024). However, the study also had limitations. As an ecological study, it suggested associations between PM_2.5_ components and SCZ but could not establish causality. Additionally, using city‐level average exposure to assess PM_2.5_ components failed to capture local spatial variation. Variables like greenery extent and socio‐economic level could impact the findings and necessitate additional consideration and adjustment. Furthermore, the study did not thoroughly explore the interaction mechanisms among PM_2.5_ components, necessitating the development of more suitable statistical methods to comprehensively evaluate their combined exposure effects in future research.

## 5 Conclusions

The research indicates that brief contact with BC and OM constituents in PM_2.5_ could pose risks for the initial appearance and relapse of SCZ. Based on this, we recommend that in areas with high concentrations of BC, OM, and SO_4_^2−^, air pollution protection measures should be strengthened, particularly for men and young to middle‐aged populations.

## Conflict of Interest

The authors declare no conflicts of interest relevant to this study.

## Supporting information

Supporting Information S1

## Data Availability

The PM_2.5_ chemical composition concentration data used in this manuscript are derived from the “Tracking Air Pollution,” which is currently under updates and can be obtained by application via the Tracking Air Pollution Team (2022) (http://tapdata.org.cn/?page_id=59&item=pm25) (Geng et al., 2017; Liu et al., 2022). The schizophrenia data were provided by The Fifth People's Hospital of Nanning under a strict data use agreement. Due to patient privacy protection laws and ethical review requirements, the data are not publicly accessible. Researchers seeking access to the schizophrenia data may request them from the hospital at nnwyyxkb@163.com. Please contact the corresponding author for further guidance on access procedures. Data analysis was performed using software (R Version 4.3.1).
