# Supplementary material for: Short‐Term Impacts of PM2.5 Components on Schizophrenia Hospitalization: A Time‐Series Analysis From Nanning, China
Source: Geohealth. 2026 Jun 7;10(6):e2025GH001556. doi: 10.1029/2025GH001556 (PMC13243193; doi:10.1029/2025GH001556)
Supplement: Supplementary file 1 — Supporting Information S1 [file GH2-10-e2025GH001556-s001.pdf]

[GeoHealth]

Supporting Information for

[Short-term Impacts of PM2.5 Components on Schizophrenia Hospitalization : a Time-series Analysis from Nanning, China]

[Zukang Gong[1, †], Baihua Chen[2, †], Bin Xu[3, †], Lan Lan[1], Shuiqing Qin[1], Li Su[2,4,*], Wenzhen lin[5,*], Jianxiong Long[2,*]]

[1 The Fifth People's Hospital of Nanning City, Nanning, China, 530001

2 School of Public Health, Guangxi Medical University, Nanning, China, 530021

3 Nanning Center for Disease Control and Prevention, Nanning, China, 530028

4 China (Guangxi) - ASEAN Engineering Research Center of Big Data for Public Health , Guangxi Medical University, Nanning, 530021, China

5 Department of Biochemistry and Molecular Biology, School of Basic Medical Sciences, Guangxi Medical University, Nanning, 530021, China

†: First co-author

*: Corresponding author]

Contents of this file

Figures S1 to S2
Tables S1 to S8

Additional Supporting Information

Captions for Tables S1 to S8
Table S1: Spearman’s correlations between PM2.5 components and weather factors.
Table S2: Single-day lag and cumulative lag effects of PM2.5 and its five components.
Table S3: Single-day and Cumulative Lag Effects of Five Components relative proportions : RR and 95% CI for Associations with SCZ Hospitalization.
Table S4 Single-day lag of PM2.5 and its five components, stratified by gender.
Table S5 Single-day lag of PM2.5 and its five components, stratified by age group.

Table S6 Single-day lag of PM2.5 and its five components, stratified by season.
Table S7 The single day value of risk and 95% confidence interval in SCZ admissions on lag 4, associated with reference levels of PM2.5 and its five components increasing across different degree of freedom.
Table S8 The single day value of risk and 95% confidence interval in SCZ admissions on lag 4, associated with reference levels of PM2.5 and its five components increasing across different degree of freedom.

Captions for Figure S1 to S2
Figure S1 Temporal trends of SCZ hospital admissions, PM2.5 and its components, and meteorological factors in Nanning (2014 - 2023).
Figure S2 Relative risk (RR) and 95% CI for the relationship between PM2.5 components exposure and SCZ hospitalization adjusted for total PM2.5.

Introduction

- This Supporting Information provides supplementary materials to the main manuscript, including eight tables (Table S1–S8) and two figures (Figure S1–S2). Table S1 presents the correlation analysis results between $PM_{2.5}$, its five chemical components, temperature, and relative humidity. Tables S2–S3 outline the lag effects of $PM_{2.5}$ components and their relative changes. Tables S4–S6 detail the results of subgroup analyses. Tables S7–S8 display the statistical outcomes after adjusting for degrees of freedom. Figure S1 visualizes the temporal variation trends of $PM_{2.5}$, its five chemical components, temperature, and relative humidity. Figure S2 presents the analytical results after adjusting for total $PM_{2.5}$ concentration.

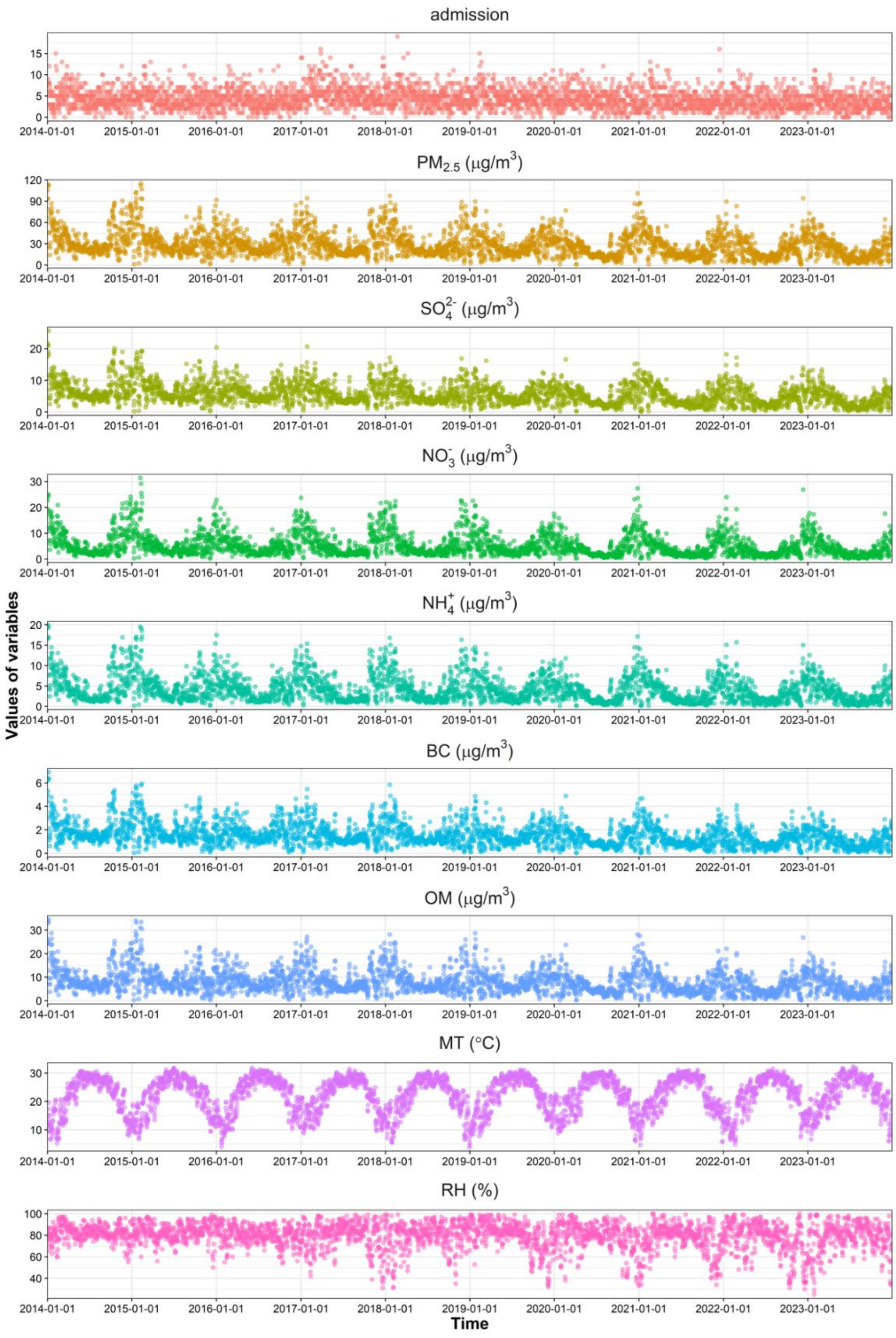

Figure S1. Temporal trends of SCZ hospital admissions, $PM_{2.5}$ and its components, and meteorological factors in Nanning (2014 - 2023)

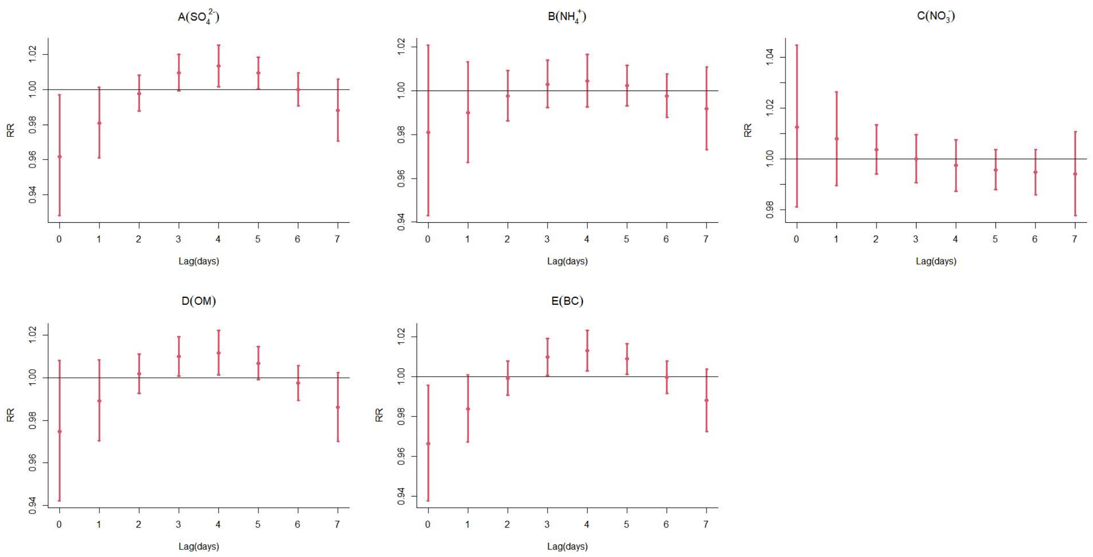

Figure S2 Relative risk (RR) and 95% CI for the relationship between $PM_{2.5}$ components exposure and SCZ hospitalization adjusted for total $PM_{2.5}$.

Table S1. Spearman's correlations between PM2.5 components and weather factors.

Variable	$PM_{2.5}$	BC	NH_4^+	NO_3^-	OM	SO_4^{2-}	MT	RH
$PM_{2.5}$	1							
BC	0.93**	1						
NH_4^+	0.94**	0.83**	1					
NO_3^-	0.94**	0.80**	0.98**	1				
OM	0.97**	0.97**	0.89**	0.88**	1			
SO_4^{2-}	0.96**	0.93**	0.92**	0.89**	0.94**	1		
MT	−0.35**	−0.17**	−0.51**	−0.53**	−0.24**	−0.30**	1	
RH	−0.32**	−0.31**	−0.30**	−0.28**	−0.35**	−0.23**	−0.002	1

Abbreviations and notes: SO42-, sulfate; NO3-, nitrate; NH4+, ammonium; OM, organic matter; BC, carbon black; MT, mean temperature; RH, relative humidity; **, P<0.01.

Table S2. Single-day lag and cumulative lag effects of PM2.5 and its five components

variable	lagday	RR	cRR
PM2.5	lag0	0.985 (0.968, 1.004)	0.985 (0.968, 1.004)
	lag1	0.995 (0.984, 1.005)	0.980 (0.953, 1.008)
	lag2	1.002 (0.995, 1.010)	0.983 (0.952, 1.014)
	lag3	1.007 (0.998, 1.017)	0.990 (0.958, 1.023)
	lag4	1.008 (0.998, 1.018)	0.998 (0.963, 1.034)
	lag5	1.004 (0.997, 1.012)	1.002 (0.966, 1.041)
	lag6	0.998 (0.989, 1.007)	1.000 (0.962, 1.040)
	lag7	0.990 (0.973, 1.007)	0.990 (0.948, 1.034)
SO4	lag0	0.980 (0.961, 0.999)	0.980 (0.961, 0.999)
	lag1	0.991 (0.981, 1.002)	0.971 (0.943, 1.001)
	lag2	1.002 (0.993, 1.010)	0.973 (0.941, 1.006)

	lag3	1.008 (0.998, 1.019)	0.981 (0.947, 1.016)
	lag4	1.010 (1.000, 1.020)	0.991 (0.954, 1.029)
	lag5	1.006 (0.999, 1.014)	0.997 (0.958, 1.038)
	lag6	0.999 (0.990, 1.009)	0.996 (0.956, 1.038)
	lag7	0.991 (0.974, 1.008)	0.987 (0.943, 1.034)
NH4+	lag0	0.987 (0.968, 1.006)	0.987 (0.968, 1.006)
	lag1	0.994 (0.983, 1.004)	0.981 (0.952, 1.010)
	lag2	0.999 (0.991, 1.008)	0.980 (0.948, 1.013)
	lag3	1.003 (0.992, 1.014)	0.983 (0.949, 1.018)
	lag4	1.004 (0.993, 1.014)	0.986 (0.950, 1.024)
	lag5	1.002 (0.994, 1.010)	0.988 (0.949, 1.028)
	lag6	0.998 (0.988, 1.008)	0.985 (0.945, 1.028)
	lag7	0.993 (0.975, 1.011)	0.978 (0.934, 1.025)
NO3-	lag0	0.997 (0.980, 1.013)	0.997 (0.980, 1.013)
	lag1	0.998 (0.989, 1.008)	0.995 (0.970, 1.020)
	lag2	1.000 (0.993, 1.007)	0.995 (0.967, 1.024)
	lag3	1.000 (0.991, 1.010)	0.995 (0.966, 1.026)
	lag4	1.000 (0.990, 1.009)	0.995 (0.963, 1.028)
	lag5	0.998 (0.990, 1.005)	0.992 (0.958, 1.028)
	lag6	0.995 (0.986, 1.003)	0.987 (0.951, 1.024)
	lag7	0.991 (0.976, 1.007)	0.978 (0.939, 1.020)
BC	lag0	0.980 (0.963, 0.997)	0.980 (0.963, 0.997)
	lag1	0.992 (0.982, 1.001)	0.972 (0.946, 0.998)
	lag2	1.002 (0.995, 1.009)	0.974 (0.945, 1.003)
	lag3	1.009 (1.000, 1.018)	0.983 (0.953, 1.013)
	lag4	1.010 (1.001, 1.019)*	0.993 (0.961, 1.025)
	lag5	1.007 (1.000, 1.013)*	0.999 (0.966, 1.034)
	lag6	0.999 (0.991, 1.007)	0.999 (0.965, 1.034)
	lag7	0.990 (0.975, 1.006)	0.989 (0.951, 1.028)
OM	lag0	0.983 (0.967, 1.000)	0.983 (0.967, 1.000)
	lag1	0.994 (0.985, 1.004)	0.978 (0.953, 1.003)
	lag2	1.004 (0.997, 1.011)	0.981 (0.954, 1.010)
	lag3	1.010 (1.001, 1.019)*	0.991 (0.962, 1.021)
	lag4	1.010 (1.001, 1.019)*	1.001 (0.970, 1.033)
	lag5	1.006 (0.999, 1.012)	1.007 (0.974, 1.040)
	lag6	0.998 (0.989, 1.006)	1.004 (0.970, 1.039)
	lag7	0.988 (0.973, 1.003)	0.992 (0.954, 1.031)

Note: 1. SO_4^{2-}, Sulfate; NO_3^-, Nitrate; NH_4^+, Ammonium; OM, Organic matter; BC, Carbon black. *: P<0.05. RR: relative risk; cRR: cumulative relative risk. 2. The RR represent changes in SCZ hospitalization per inter-quartile range (IQR) $\mu g/m^3$ increase in $PM_{2.5}$ components. 2. SO_4^{2-}: Sulfate; NO_3^-: Nitrate: NH_4^+, Ammonium: OM, Organic matter; BC: Carbon black. *: P<0.05.

Table S3. Single-day and Cumulative Lag Effects of Five Components relative proportions : RR and 95% CI for Associations with SCZ Hospitalization.

variable	lagday	RR	cRR
SO_4^{2-} RP	lag0	0.988 (0.969, 1.008)	0.988 (0.969, 1.008)
	lag1	0.993 (0.982, 1.005)	0.981 (0.952, 1.012)
	lag2	0.998 (0.989, 1.006)	0.979 (0.946, 1.014)
	lag3	1.001 (0.990, 1.012)	0.980 (0.945, 1.017)
	lag4	1.002 (0.991, 1.013)	0.982 (0.944, 1.021)
	lag5	1.001 (0.993, 1.009)	0.983 (0.943, 1.024)
	lag6	0.998 (0.988, 1.008)	0.981 (0.940, 1.024)
	lag7	0.995 (0.977, 1.013)	0.976 (0.931, 1.024)
NH_4^+ RP	lag0	0.996 (0.970, 1.023)	0.996 (0.970, 1.023)
	lag1	0.996 (0.981, 1.012)	0.993 (0.953, 1.034)
	lag2	0.996 (0.985, 1.008)	0.989 (0.943, 1.037)
	lag3	0.996 (0.982, 1.010)	0.985 (0.936, 1.037)
	lag4	0.996 (0.982, 1.010)	0.981 (0.929, 1.036)
	lag5	0.996 (0.984, 1.007)	0.977 (0.921, 1.036)
	lag6	0.995 (0.981, 1.009)	0.972 (0.914, 1.033)
	lag7	0.995 (0.971, 1.019)	0.967 (0.903, 1.035)
NO_3^- RP	lag0	1.017 (0.990, 1.044)	1.017 (0.990, 1.044)
	lag1	1.006 (0.990, 1.022)	1.023 (0.981, 1.066)
	lag2	0.997 (0.986, 1.008)	1.019 (0.972, 1.069)
	lag3	0.990 (0.977, 1.003)	1.009 (0.960, 1.060)
	lag4	0.987 (0.974, 1.000)	0.995 (0.945, 1.049)
	lag5	0.987 (0.977, 0.998)*	0.983 (0.931, 1.038)
	lag6	0.991 (0.978, 1.003)	0.973 (0.920, 1.029)
	lag7	0.995 (0.973, 1.018)	0.968 (0.910, 1.030)
BC RP	lag0	0.997 (0.981, 1.013)	0.997 (0.981, 1.013)
	lag1	1.000 (0.991, 1.009)	0.997 (0.973, 1.022)
	lag2	1.003 (0.996, 1.01)	1.000 (0.973, 1.029)
	lag3	1.005 (0.996, 1.013)	1.005 (0.975, 1.036)
	lag4	1.005 (0.996, 1.013)	1.010 (0.978, 1.043)
	lag5	1.003 (0.997, 1.01)	1.013 (0.979, 1.048)
	lag6	1.000 (0.992, 1.009)	1.014 (0.978, 1.050)
	lag7	0.997 (0.983, 1.011)	1.011 (0.972, 1.051)
OM RP	lag0	1.001 (0.985, 1.017)	1.001 (0.985, 1.017)
	lag1	1.007 (0.998, 1.016)	1.008 (0.983, 1.033)
	lag2	1.011 (1.004, 1.018)*	1.019 (0.991, 1.049)
	lag3	1.013 (1.005, 1.022)*	1.033 (1.003, 1.064)*
	lag4	1.012 (1.003, 1.021)*	1.045 (1.013, 1.079)*
	lag5	1.007 (1.000, 1.013)	1.052 (1.017, 1.088)*
	lag6	0.999 (0.991, 1.007)	1.051 (1.015, 1.088)*
	lag7	0.990 (0.976, 1.004)	1.040 (1.001, 1.081)*

Abbreviations: RP, relative proportions; $PM_{2.5}$, SO_4^{2-}, sulfate: NO_3^-; nitrate, NH_4^+, ammonium; OM, organic matter; BC, carbon black; *: $P<0.05$; cRR: cumulative RR.

Notes: RR represents changes in SCZ hospitalization risk per inter-quartile range (IQR) increase in $PM_{2.5}$ components relative proportions.

Table S4. Single-day lag of PM2.5 and its five components, stratified by gender.

Variable ($\mu g/m^3$)	Lag (day)	*RR*(95%*CI*)	
		Male	Female
$PM_{2.5}$	lag0	0.975 (0.950, 1.000)	0.997 (0.971, 1.023)
	lag1	0.990 (0.976, 1.004)	0.997 (0.958, 1.037)
	lag2	1.003 (0.992, 1.014)	0.999 (0.955, 1.044)
	lag3	1.012 (0.998, 1.026)	1.002 (0.956, 1.050)
	lag4	1.014 (1.000, 1.028)*	1.004 (0.955, 1.055)
	lag5	1.010 (1.000, 1.021)	1.002 (0.950, 1.057)
	lag6	1.002 (0.989, 1.015)	0.996 (0.943, 1.053)
	lag7	0.991 (0.968, 1.015)	0.985 (0.926, 1.049)
SO_4^{2-}	lag0	0.968 (0.942, 0.995)	0.992 (0.965, 1.020)
	lag1	0.987 (0.972, 1.002)	0.996 (0.981, 1.012)
	lag2	1.003 (0.991, 1.015)	1.000 (0.989, 1.012)
	lag3	1.014 (1.000, 1.029)	1.002 (0.988, 1.017)
	lag4	1.018 (1.004, 1.032)*	1.002 (0.987, 1.016)
	lag5	1.014 (1.003, 1.025)*	0.999 (0.988, 1.010)
	lag6	1.005 (0.992, 1.018)	0.994 (0.981, 1.008)
	lag7	0.993 (0.969, 1.017)	0.989 (0.965, 1.014)
NH_4^+	lag0	0.973 (0.947, 1.000)	1.002 (0.974, 1.030)
	lag1	0.988 (0.973, 1.003)	1.000 (0.984, 1.015)
	lag2	1.000 (0.989, 1.012)	0.998 (0.986, 1.010)
	lag3	1.009 (0.994, 1.024)	0.997 (0.981, 1.012)
	lag4	1.011 (0.996, 1.027)	0.996 (0.981, 1.011)
	lag5	1.008 (0.996, 1.019)	0.995 (0.984, 1.007)
	lag6	1.000 (0.986, 1.014)	0.995 (0.981, 1.010)
	lag7	0.990 (0.965, 1.016)	0.996 (0.970, 1.022)
NO_3^-	lag0	0.983 (0.960, 1.006)	1.011 (0.987, 1.035)
	lag1	0.992 (0.979, 1.005)	1.005 (0.992, 1.018)
	lag2	1.000 (0.989, 1.010)	1.000 (0.990, 1.010)
	lag3	1.005 (0.991, 1.018)	0.996 (0.983, 1.009)
	lag4	1.006 (0.992, 1.019)	0.993 (0.980, 1.007)
	lag5	1.002 (0.992, 1.013)	0.993 (0.982, 1.003)
	lag6	0.996 (0.984, 1.009)	0.993 (0.980, 1.005)
	lag7	0.989 (0.967, 1.012)	0.994 (0.971, 1.017)
BC	lag0	0.974 (0.95, 0.9980)	0.987 (0.962, 1.011)
	lag1	0.989 (0.976, 1.003)	0.995 (0.981, 1.009)
	lag2	1.003 (0.993, 1.013)	1.002 (0.992, 1.012)
	lag3	1.012 (0.999, 1.025)	1.006 (0.993, 1.019)
	lag4	1.015 (1.002, 1.028)*	1.006 (0.993, 1.019)
	lag5	1.011 (1.002, 1.02 0)*	1.002 (0.993, 1.011)
	lag6	1.003 (0.992, 1.015)	0.995 (0.984, 1.007)

	lag7	0.993 (0.972, 1.015)	0.987 (0.966, 1.009)
OM	lag0	0.974 (0.951, 0.997)	0.994 (0.970, 1.018)
	lag1	0.990 (0.977, 1.003)	0.999 (0.986, 1.012)
	lag2	1.004 (0.994, 1.014)	1.003 (0.994, 1.013)
	lag3	1.013 (1.001, 1.026)*	1.006 (0.993, 1.019)
	lag4	1.015 (1.003, 1.028)*	1.005 (0.992, 1.018)
	lag5	1.010 (1.001, 1.019)*	1.001 (0.992, 1.011)
	lag6	1.000 (0.989, 1.011)	0.995 (0.984, 1.007)
	lag7	0.987 (0.966, 1.009)	0.988 (0.967, 1.010)

Note: 1. SO_4^{2-}, Sulfate; NO_3^-, Nitrate; NH_4^+, Ammonium; OM, Organic matter; BC, Carbon black. *: P<0.05. 2. The relative risk (RR) represent changes in SCZ hospitalization per inter-quartile range (IQR) μg/m^3 increase in $PM_{2.5}$ components.

Table S5. Single-day lag of PM2.5 and its five components, stratified by age group.

Variable (μg/m3)	Lag (day)	RR	
		Age<45	Age>=45
$PM_{2.5}$	lag0	0.981 (0.960, 1.002)	0.997 (0.963, 1.032)
	lag1	0.993 (0.981, 1.005)	0.999 (0.980, 1.018)
	lag2	1.003 (0.994, 1.013)	1.000 (0.985, 1.015)
	lag3	1.011 (0.999, 1.022)	0.999 (0.981, 1.018)
	lag4	1.013 (1.001, 1.025)*	0.996 (0.978, 1.015)
	lag5	1.010 (1.001, 1.018)*	0.991 (0.977, 1.005)
	lag6	1.003 (0.992, 1.014)	0.984 (0.967, 1.001)
	lag7	0.995 (0.975, 1.015)	0.977 (0.946, 1.008)
SO_4^{2-}	lag0	0.979 (0.957, 1.001)	0.982 (0.946, 1.019)
	lag1	0.992 (0.979, 1.005)	0.990 (0.969, 1.011)
	lag2	1.003 (0.994, 1.013)	0.997 (0.981, 1.013)
	lag3	1.011 (0.999, 1.023)	1.001 (0.981, 1.020)
	lag4	1.014 (1.002, 1.026)*	1.000 (0.981, 1.019)
	lag5	1.011 (1.002, 1.020)*	0.994 (0.980, 1.009)
	lag6	1.005 (0.994, 1.016)	0.986 (0.968, 1.004)
	lag7	0.997 (0.976, 1.017)	0.976 (0.944, 1.009)
NH_4^+	lag0	0.984 (0.962, 1.006)	0.995 (0.959, 1.032)
	lag1	0.992 (0.980, 1.005)	0.997 (0.976, 1.018)
	lag2	1.000 (0.990, 1.009)	0.998 (0.982, 1.014)
	lag3	1.005 (0.992, 1.018)	0.998 (0.977, 1.018)
	lag4	1.007 (0.994, 1.020)	0.995 (0.975, 1.016)
	lag5	1.006 (0.996, 1.016)	0.990 (0.975, 1.006)
	lag6	1.003 (0.991, 1.015)	0.984 (0.966, 1.003)
	lag7	0.999 (0.977, 1.020)	0.978 (0.945, 1.012)
NO_3^-	lag0	0.991 (0.971, 1.010)	1.011 (0.980, 1.043)
	lag1	0.996 (0.985, 1.007)	1.005 (0.988, 1.023)
	lag2	1.000 (0.991, 1.009)	0.999 (0.986, 1.013)
	lag3	1.003 (0.992, 1.014)	0.994 (0.977, 1.012)
	lag4	1.004 (0.993, 1.015)	0.989 (0.972, 1.007)

	lag5	1.002 (0.994, 1.011)	0.985 (0.972, 0.999)
	lag6	1.000 (0.990, 1.010)	0.981 (0.965, 0.998)
	lag7	0.997 (0.978, 1.016)	0.978 (0.949, 1.008)
BC	lag0	0.978 (0.958, 0.998)	0.985 (0.953, 1.019)
	lag1	0.991 (0.980, 1.002)	0.994 (0.976, 1.012)
	lag2	1.002 (0.994, 1.011)	1.001 (0.988, 1.014)
	lag3	1.011 (1.000, 1.021)	1.005 (0.988, 1.022)
	lag4	1.013 (1.003, 1.024)*	1.003 (0.986, 1.020)
	lag5	1.011 (1.003, 1.018)*	0.996 (0.984, 1.009)
	lag6	1.004 (0.995, 1.014)	0.986 (0.971, 1.001)
	lag7	0.996 (0.978, 1.015)	0.975 (0.947, 1.003)
OM	lag0	0.980 (0.960, 0.999)*	0.993 (0.962, 1.025)
	lag1	0.993 (0.982, 1.004)	0.998 (0.981, 1.016)
	lag2	1.004 (0.996, 1.012)	1.003 (0.990, 1.016)
	lag3	1.012 (1.001, 1.022)*	1.004 (0.987, 1.021)
	lag4	1.014 (1.003, 1.025)*	1.001 (0.984, 1.018)
	lag5	1.010 (1.003, 1.018)*	0.993 (0.981, 1.006)
	lag6	1.003 (0.994, 1.013)	0.983 (0.968, 0.998)
	lag7	0.994 (0.976, 1.013)	0.971 (0.943, 1.000)

Note: 1. SO_4^{2-}, Sulfate; NO_3^-, Nitrate; NH_4^+, Ammonium; OM, Organic matter; BC, Carbon black. *: P<0.05. 2. The relative risk (RR) represent changes in SCZ hospitalization per inter-quartile range (IQR) μg/m³ increase in $PM_{2.5}$ components. 2. SO_4^{2-}: Sulfate; NO_3^-: Nitrate: NH_4^+, Ammonium: OM, Organic matter; BC: Carbon black. *: P<0.05.

Table S6. Single-day lag of PM2.5 and its five components, stratified by season.

Variable (μg/m3)	Lag (day)	RR	
		Cold season	Warm season
$PM_{2.5}$	lag0	0.993 (0.972, 1.015)	0.957 (0.917, 0.998)
	lag1	0.997 (0.985, 1.010)	0.984 (0.961, 1.007)
	lag2	1.001 (0.992, 1.010)	1.007 (0.990, 1.025)
	lag3	1.003 (0.992, 1.014)	1.024 (1.001, 1.047)*
	lag4	1.003 (0.992, 1.014)	1.028 (1.005, 1.052)*
	lag5	1.001 (0.992, 1.009)	1.021 (1.003, 1.040)*
	lag6	0.997 (0.987, 1.008)	1.007 (0.984, 1.030)
	lag7	0.993 (0.974, 1.013)	0.988 (0.950, 1.029)
SO_4^{2-}	lag0	0.989 (0.966, 1.013)	0.957 (0.919, 0.996)
	lag1	0.995 (0.981, 1.009)	0.984 (0.962, 1.006)
	lag2	0.999 (0.989, 1.010)	1.008 (0.991, 1.025)
	lag3	1.003 (0.991, 1.015)	1.024 (1.003, 1.047)*
	lag4	1.004 (0.992, 1.015)	1.029 (1.007, 1.051)*
	lag5	1.002 (0.993, 1.011)	1.021 (1.004, 1.038)*
	lag6	0.999 (0.988, 1.011)	1.005 (0.984, 1.027)
	lag7	0.996 (0.976, 1.016)	0.986 (0.949, 1.024)
NH_4^+	lag0	0.992 (0.970, 1.015)	0.961 (0.921, 1.004)
	lag1	0.995 (0.982, 1.008)	0.983 (0.959, 1.007)

	lag2	0.997 (0.987, 1.007)	1.003 (0.984, 1.022)
	lag3	0.998 (0.986, 1.010)	1.017 (0.993, 1.042)
	lag4	0.998 (0.986, 1.010)	1.023 (0.999, 1.048)
	lag5	0.997 (0.987, 1.006)	1.021 (1.001, 1.040)*
	lag6	0.994 (0.983, 1.006)	1.012 (0.988, 1.037)
	lag7	0.992 (0.971, 1.013)	1.002 (0.960, 1.045)
NO_3^-	lag0	1.001 (0.983, 1.020)	0.957 (0.914, 1.001)
	lag1	0.999 (0.989, 1.010)	0.982 (0.956, 1.008)
	lag2	0.998 (0.990, 1.006)	1.004 (0.984, 1.026)
	lag3	0.996 (0.986, 1.007)	1.021 (0.995, 1.048)
	lag4	0.995 (0.984, 1.005)	1.028 (1.002, 1.055)*
	lag5	0.993 (0.985, 1.001)	1.025 (1.004, 1.047)*
	lag6	0.992 (0.982, 1.002)	1.016 (0.989, 1.043)
	lag7	0.990 (0.973, 1.008)	1.003 (0.959, 1.050)
BC	lag0	0.983 (0.962, 1.004)	0.975 (0.940, 1.011)
	lag1	0.993 (0.981, 1.005)	0.990 (0.971, 1.011)
	lag2	1.002 (0.993, 1.010)	1.003 (0.989, 1.018)
	lag3	1.007 (0.997, 1.018)	1.012 (0.993, 1.032)
	lag4	1.009 (0.998, 1.019)	1.015 (0.995, 1.035)
	lag5	1.006 (0.998, 1.013)	1.011 (0.996, 1.026)
	lag6	1.000 (0.990, 1.010)	1.002 (0.984, 1.022)
	lag7	0.992 (0.974, 1.011)	0.992 (0.959, 1.026)
OM	lag0	0.992 (0.970, 1.015)	0.965 (0.928, 1.003)
	lag1	0.995 (0.982, 1.008)	0.988 (0.967, 1.009)
	lag2	0.997 (0.987, 1.007)	1.008 (0.992, 1.024)
	lag3	0.998 (0.986, 1.010)	1.022 (1.001, 1.042)*
	lag4	0.998 (0.986, 1.010)	1.025 (1.004, 1.046)*
	lag5	0.997 (0.987, 1.006)	1.017 (1.001, 1.034)*
	lag6	0.994 (0.983, 1.006)	1.003 (0.983, 1.023)
	lag7	0.992 (0.971, 1.013)	0.986 (0.951, 1.022)

Note: 1. SO_4^{2-}, Sulfate; NO_3^-, Nitrate; NH_4^+, Ammonium; OM, Organic matter; BC, Carbon black. *: $P<0.05$. 2. The relative risk (RR) represent changes in SCZ hospitalization per inter-quartile range (IQR) μg/m^3 increase in $PM_{2.5}$ components.

Table S7. The single day value of risk and 95% confidence interval in SCZ admissions on lag 4, associated with reference levels of PM2.5 and its five components increasing across different degree of freedom.

Degree of freedom	$PM_{2.5}$	SO_4^{2+}	NO_3^-	NH_4^+	OM	BC
df=1	1.008 (0.998, 1.018)	1.010 (1.000, 1.02)	1.000 (0.991, 1.009)	1.004 (0.993, 1.015)	1.011 (1.002, 1.02)	1.011 (1.002, 1.02)
df=2	1.008 (0.998, 1.018)	1.010 (1, 1.02)	1.000 (0.990, 1.009)	1.004 (0.993, 1.014)	1.01 (1.001, 1.019)	1.011 (1.002, 1.02)
df=3	1.008 (0.998, 1.018)	1.010 (1.000, 1.020)	1.000 (0.990, 1.009)	1.004 (0.993, 1.014)	1.010 (1.001, 1.019)	1.010 (1.001, 1.019)
df=4	1.008 (0.999, 1.018)	1.010 (1.000, 1.02)	1.000 (0.991, 1.010)	1.004 (0.993, 1.015)	1.01 (1.001, 1.02)	1.01 (1.001, 1.02)
df=5	1.009 (0.999, 1.019)	1.010 (1.000, 1.021)	1.000 (0.991, 1.010)	1.004 (0.994, 1.015)	1.011 (1.002, 1.02)	1.011 (1.002, 1.02)

Note: Adjust df for MT and RH from 1 to 5

Table S8. The single day value of risk and 95% confidence interval in SCZ admissions on lag 4, associated with reference levels of PM2.5 and its five components increasing across different degree of freedom.

Degree of freedom	$PM_{2.5}$	SO_4^{2+}	NO_3^-	NH_4^+	OM	BC
df=5	1.009 (0.999, 1.019)	1.011 (1.001, 1.021)	1.001 (0.991, 1.010)	1.005 (0.994, 1.016)	1.011 (1.002, 1.020)	1.011 (1.002, 1.021)
df=6	1.009 (0.999, 1.019)	1.011 (1.000, 1.021)	1.000 (0.991, 1.010)	1.005 (0.994, 1.015)	1.011 (1.002, 1.020)	1.011 (1.002, 1.02)
df=7	1.008 (0.998, 1.018)	1.010 (1.000, 1.020)	1.000 (0.990, 1.009)	1.004 (0.993, 1.014)	1.010 (1.001, 1.019)	1.010 (1.001, 1.019)
df=8	1.008 (0.998, 1.018)	1.010 (1.000, 1.02)	0.999 (0.990, 1.009)	1.003 (0.993, 1.014)	1.010 (1.001, 1.019)	1.010 (1.001, 1.019)
df=9	1.008 (0.998, 1.018)	1.010 (1.000, 1.02)	0.999 (0.990, 1.009)	1.003 (0.993, 1.014)	1.010 (1.001, 1.019)	1.010 (1.001, 1.019)

Note: Adjust df for time from 7 to 9
